# Periosteum coverage versus collagen-membrane coverage in periodontally accelerated osteogenic orthodontics: a randomized controlled clinical trial in Class II and Class III malocclusions

**DOI:** 10.1186/s12903-022-02477-8

**Published:** 2022-10-08

**Authors:** Zhigui Ma, Yan Zhu, Yining Zhan, Yufan Zhang, Ahmed Abdelrehem, Bian Wang, Chi Yang

**Affiliations:** 1 Department of Oral Surgery, Shanghai Ninth People’s Hospital, College of Stomatology, Shanghai Jiao Tong University School of Medicine, National Clinical Research Center for Oral Diseases, Shanghai Key Laboratory of Stomatology & Shanghai Research Institute of Stomatology, No. 639, Zhizaoju Road, Shanghai, 200001 People’s Republic of China; 2 Department of Craniomaxillofacial and Plastic Surgery, Faculty of Dentistry, Alexandria University, Alexandria, Egypt; 3 Department of Assisted Reproduction, Shanghai Ninth People’s Hospital Affiliated With Jiao Tong University, School of Medicine, No. 639, Zhizaoju Road, Shanghai, 200001 People’s Republic of China

**Keywords:** Alveolar bone defect, Bone regenerative surgery, Periosteum, Collagen membrane, Vertical bone augmentation

## Abstract

**Background:**

Periodontal accelerated osteogenic orthodontics (PAOO) is a widely-used clinical procedure that combines selective alveolar corticotomy, particulate bone grafting, and the application of orthodontic forces. Different modifications of PAOO such as collagen-membrane coverage can better benefit patients from preventing displacement of grafts. Due to its stability, collagen-membrane coverage gradually gained popularity and became a widely-used procedure in traditional PAOO technique.

**Objectives:**

To quantitatively investigate the radiographic changes of alveolar bone, periodontal soft tissue changes of the mandibular anterior teeth and postoperative complications in periosteum-covered techniques compared with traditional surgical technique in PAOO.

**Methods:**

Orthodontic camouflage for dental Class II or decompensation for skeletal Class III malocclusions were included; Patients with bone defects on the buccal aspects of the anterior mandible regions confirmed by clinical and radiographic examination were randomly divided into the periosteum coverage group or traditional technique group for PAOO. Cone-beam computerized tomography (CBCT) scans were obtained before treatment (T0) and 1 week (T1) and 12 months (T2) after operation. The primary outcome variable was the vertical alveolar bone level (VBL), the secondary evaluation parameters included labial horizontal bone thickness at the midpoint of the middle third (MHBT) or apical third (AHBT) to the limit of the labial cortical surface during a 12-month follow-up. Postoperative sequelae were evaluated after 2 days and 7 days in both the groups. Periodontal parameters were analyzed at T0 and T2.

**Results:**

Thirty-six adult subjects were eligible and recruited in the present study. Although experimental group exhibited more severe infection, no significant differences of the postoperative symptoms or periodontal parameters was found between the 2 groups (*P* > 0.05). All patients were examined respectively using CBCT at baseline (T0), postoperative 1 week (T1) and 12 months (T2). Both alveolar bone height and width increased from T0 to T1 (*P* < 0.001) and then reduced from T1 to T2 (*P* < 0.001) in both groups. However, significant bone augmentation was achieved in each group from T0 to T2 (*P* < 0.001). Furthermore, the vertical alveolar bone augmentation in the experimental group increased significantly than that in the traditional surgery (*P* < 0.05).

**Conclusions:**

Compared with traditional PAOO surgery, the periosteum-covered technique provides superior graft stabilization and satisfactory vertical bone augmentation in the labial mandibular anterior area.

**Supplementary Information:**

The online version contains supplementary material available at 10.1186/s12903-022-02477-8.

## Background

Orthodontic tooth movement is based on the remodeling of the alveolar bone by mechanical force. Labial inclination of the mandibular incisors is often performed for patients with orthodontic camouflage treatment in Class II or dental decompensation in skeletal Class III deformities. According to researches, pre-existing alveolar defect is more commonly in dolichofacial individuals [1, 2].There was a definite limit for tooth movement as the apex abuts the cortical plates of the alveolus, which could be considered as “orthodontic walls” [3]. From clinical observation, challenging these boundaries may lead to thinner bone thickness at labial side and gingival recessions [2, 4]. Therefore, some researchers advocated that tooth movement should be limited to prevent iatrogenic sequelae [3]. However, inadequately proclination will compromise the quality and quantity of camouflage or surgical correction and thus can limit the ability to achieve ideal outcomes. It was considered as a contradiction in orthodontic treatment that patients who presented with alveolar deficiency are inadequate to the demand of extensive tooth movement.

PAOO has been proposed to solve many limitations in the orthodontic treatment of adults such as accelerating tooth movement and supporting alveolar bone thickness [5, 6].The innovative technique combined the refined corticotomy-facilitated orthodontic treatment and bone regeneration, including selective decortication, bone-grafting implantation, and the application of orthodontic forces. The increased alveolar volume provides bone support for both teeth and the periodontal tissues, thereby improve gingival bio-type. Several reports indicated that this technique was safe, efficient and might reduce the need for orthognathic surgery or teeth extraction [7–9]. In addition, the postoperative reactions such as swelling, pain and discomfort brought by PAOO operation are relatively mild [10]. Although PAOO can be considered an effective treatment approach in adult, there were still some periodontal adverse effects, including loss of interdental papillae or attached gingival [11, 12]. In addition, the regeneration of coronal and vertical alveolar bone remains a challenge with the traditional PAOO technique. It was reported that alveolar volume for PAOO at the middle and apical portions was greater than the coronal part [13]. Another study also reported the vertical alveolar bone level was reduced after PAOO in skeletal Class III patients [9].

To overcome these shortcomings, we have reported a novel surgical technique by using the periosteum for the coverage of the grafts [14]. The periosteum was fixed on the surface of the alveolar bone with sutures to prevent the grafting materials from displacement. It is still unknown whether PAOO with the periosteum or collagen membranes could effectively improve alveolus regeneration, especially for the vertical bone augmentation. In addition, many clinical researches on PAOO are often restricted by the amount of samples or lack of control group.

The aim of this study was to evaluate the effect of periodontal-covered PAOO technique on the management of mandibular anterior alveolar ridge defects in adult patients. The radiographic changes including alveolar height and thickness were quantitatively measured by CBCT. The gingival changes and postoperative complications were evaluated by clinical observations. We hypothesized that the using of periosteum-covered technique in PAOO would be more favorable compared with the conventional treatment.

## Patients and materials

### Trial design

A randomized, single-blinded, controlled trial was performed at the Department of Oral Surgery of the Ninth People’s Hospital affiliated with Shanghai Jiao Tong University School of Medicine (Shanghai, China). The trial was registered at the Chinese Clinical Trial Registry, a member of the World Health Organization international clinical trials registry (Registry Number: ChiCTR-INR-17012764). This study was approved by the ethics committee of Shanghai Ninth People’s Hospital affiliated with Shanghai Jiao Tong University, School of Medicine (Number: 2017-363-T265). All participants permitted this study and signed an informed consent agreement.

### Sample size determination

Before the initiation of the study, the power analysis for sample size calculation was performed. In short, the primary parameter is VBL. According to the preliminary results and the previous study, there is a mean VBL achievement of 2.55 mm in the experimental group and 1.48 mm (SD 1.03 mm) in the comparator group. According to the results of power analysis, a minimum of 18 patients was needed for each group so as to obtain 80% power in our study after considering 10% dropouts.

### The inclusion and exclusion criteria

Consecutive participants who presented with malocclusions requiring PAOO surgery and orthodontic treatment were recruited. The inclusion criteria included: (1) age between 18 and 30 years; (2) Labial inclination of mandibular anterior teeth for orthodontic camouflage in Class II (ANB > 4 degrees) or a decompensation in Class III malocclusion (ANB < 0 degrees); (3) thin gingival biotype assessed by the transparency of the periodontal probe through the gingival margin while probing the sulcus [15]; 4)CBCT showing dehiscence(bone defect with more than 2 mm from the cementoenamel junction (CEJ) in the cervical area) or fenestration(the defect not involve the alveolar crest) in the lower anterior regions [16, 17]; (4) anterior crowding less than 4 mm in the mandibular dental arch; (5) orthodontic appliance bonded to the teeth without archwires engagement during 1 or 2 weeks preceding the surgery (6)vertical growth pattern of hyper-divergent type(SN-MP angle > 37° assessed with CBCT). The exclusion criteria included: (1) craniofacial syndromes; (2) uncontrolled periodontal disease; (3) history of orthodontic or endodontic treatments; (4) abnormal dental morphology; (5) restorations in the mandibular anterior regions; (6) severe systemic diseases; (7) heavy smoker (more than 20 cigarettes per day); (8) antibiotic prophylaxis requirements (e.g., patients with valvular heart disease or with prosthetic joint replacements); (9) acute inflammation.

### Randomization, conceal assignment and blinding

The subjects were randomly divided to the periosteum coverage group or the collagen membrane coverage group. Researcher at the study site opened the sealed and stapled envelope with random number codes inside. Randomization sequence was created using Stata 9.0 (StataCorp, College Station, TX) statistical software. The envelopes were prepared by an individual unrelated to the clinical portion of the study. Blindness was applied during assignment of the groups and during the radiographic analysis. The trial was single-blinded, because it was not possible to mask either the surgeon or the participants as to the treatment modality, because the incision position was different in two groups.

## Interventions

### Surgical procedure

All patients received the surgery under local anesthesia with 2% lidocaine by the same surgeon (Y.C.).

**Fig. 1:**
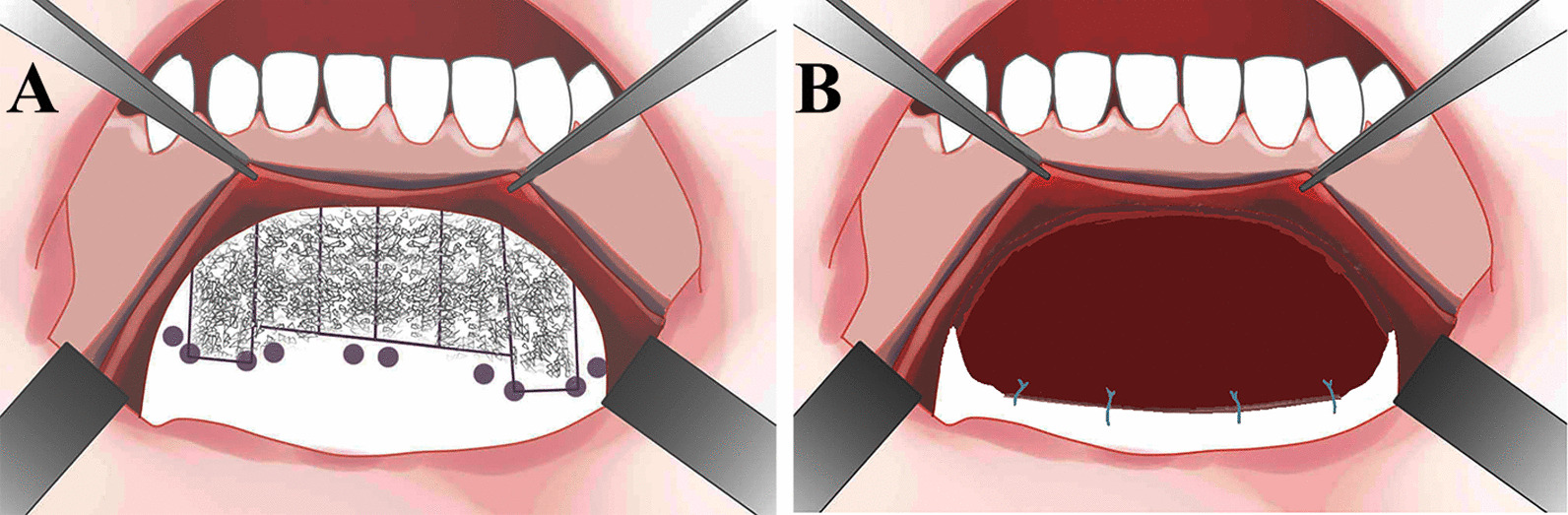
Schematic diagram of the periosteum-covered periodontally accelerated osteogenic orthodontics (PAOO) surgery. **A** Periosteum dissection, corticotomy, paired holes and grafting performed in the lower anterior region; **B** the periosteum was sutured to the alveolus through the holes and acted as an encapsulating membrane to cover the graft materials in a dumpling-like fashion

**Fig. 2:**
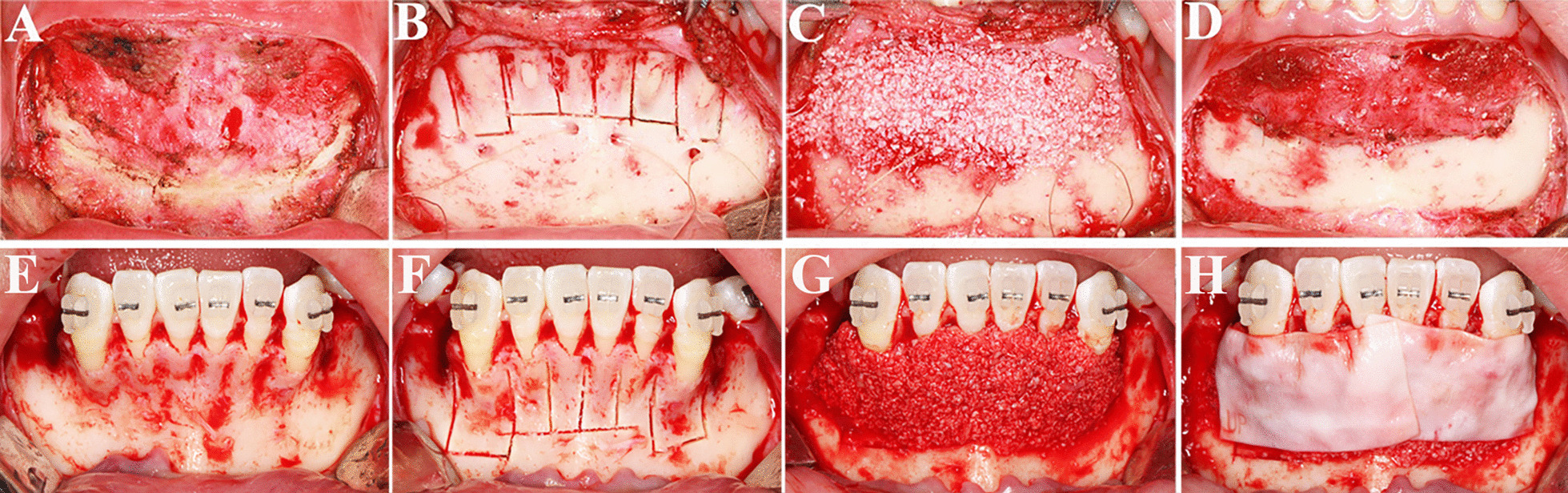
Surgical procedure. **A**–**D** PAOO with periosteum coverage; **E**–**H** PAOO with a resorbable membrane coverage

*Experimental group* According to our pervious report, the flaps were performed one tooth mesially and distally beyond the “bone activation” region, enabling full exposure of the operative field and avoiding any tension [14]. Electrosurgery has not only been widely used to cut through the soft tissue, but also used to seal off bleeding blood vessels during the whole operation. The horizontal incision at the mucogingival junction was carefully made only in the mucosa layer of the lower anterior region to ensure the integrity of the periosteum. A supraperiosteal split-thickness dissection was carefully made apically to the mental region, avoiding injury of the periosteum and enabling adequate exposure of the periosteum. The periosteum layer was given the second discission at the mental region, about 15 mm away from the initial incision, then reflected coronally. Using a small periosteal elevator, the periosteal layer was carefully raised from the alveolar surface. At the coronal portion, the periosteum was elevated slight below the peak of the crest, so as to avoid the laceration of the cervical gingival tissue. The inferior alveolar nerve should be protected while reflecting the flap. Selective alveolar corticotomy and grafting procedure were performed [14]. The periosteum was repositioned and fixed onto the surface of the alveolar bone using sutures through paired holes, which were drilled in the alveolar bone under the horizontal corticotomy (Fig. 1). In this technique, the periosteum was used as an encapsulating membrane to keep particle grafting substitutes from displacement (Fig. 2A–D).

*The comparator group* routine PAOO surgical procedure was applied in this group. After the creation of a papilla reserving incision associated with bilateral vertical releasing incisions, a full-thickness flap was reflected apically 15 mm from the CEJ [18].The bone activation was performed surrounding the anterior teeth with columnar corticotomy cuts and intramarrow penetrations. Deproteinized bovine bone mineral (Bio-Oss, Geistlich Biomateirals AG, Wolhuser, Switzerland) was laid above the activated alveolus. According to 1 cc per tooth, the total of twenty-four cubic centimeters of the bone substitutes was used. Then the graft area was covered with resorbable collagen membranes (Bio-Gide, Geistlich Biomateirals AG) and the flap was replaced and sutured (Fig. 2E–H). All participates were administrated with antibiotics for 3–5 days postoperatively, and suggested to rinse with mouthrinse three times a day for plaque control. Patients should avoid pressure over the surgical site and brushing in the grafting area for 1 week.

All subjects completed the same postoperative protocol for both groups. The protocol included antibiotic therapy (amoxicillin 50 mg/kg in 2 daily doses for 5 days) and analgesics (ibuprofen, 600 mg every 8 h) as necessary for pain control, associated with a chlorhexidine 0.2% mouthwash (3 times daily for 6 days).

### Orthodontic treatment

Self-ligating DQ brackets (DQ; Ormco, Orange, CA, USA) were used. Orthodontic tooth movement were carried out every 2 weeks after the surgical procedure; According to routine orthodontic treatment guidelines [5, 9], the archwire sequence involved 0.014-inch, 0.014 × 0.025-inch and 0.019 × 0.025-inch copper–nickel–titanium wires for aligning and leveling the arches followed by 0.019 × 0.025-inch stainless steel wires for controlling torque.

### Assessments

All participates were examined by an independent examiner who was blinded to the study group assignment and surgery performance. The examiner was well calibrated prior to the study and had 5 years of experience as an oral and maxillofacial surgeon. To better control radiographic bias, one well-trained investigator performed all measurements. Furthermore, all the results were conducted by averaging the testing values of repeated measurements.

### Operation time, swelling, and perceived pain

The operative duration was defined as the time from the incision creating to the last suture finishing. Facial swelling (increased volume of the skin) form the tragus to the pogonion distance and pain (using a 10-centmeter horizontal visual analog scale, VAS, 0 represented no pain and 10 represented the most severely pain) were assessed during the early (2 days) and late stages (7 days) of wound healing in the 2 groups [19].Postsurgical infection and neurological damage were examined for each patient.

### Outcome variables

The primary outcome variable was the VBL. The second outcome variable including the middle and the apical level of horizontal bone thickness (AHBT and AHBT, respectively), probing depth (PD), clinical attachment level (CAL), width of keratinized tissue (WKT) and postoperative complications were evaluated.

### Radiographic measurements

All patients were examined using a CBCT scanner (VG; NewTom, Verona, Italy) at baseline (T0). It was essential to take CBCT scans for identification the effect of augmentation procedures. The second image (T1) was performed 1 week after the surgery (due to preoperative preparation, this image was taken three months after T0.) and the last image (T2) was taken 12 months after the surgery [20], which could minimize exposure to radiation and was consistent with clinical guidelines for dental CBCT of Japanese Society for Oral and Maxillofacial Radiology (JSOMR) [21]. The patient’s head was oriented by positioning the Frankfort plane parallel to the horizontal plane and in centric occlusion. The imaging scanning parameters were 110 kV, 0–20 mA with an exposure time of 5.4 s and a 12-in field of view. These settings produced a voxel size of 0.125 mm.

**Fig. 3:**
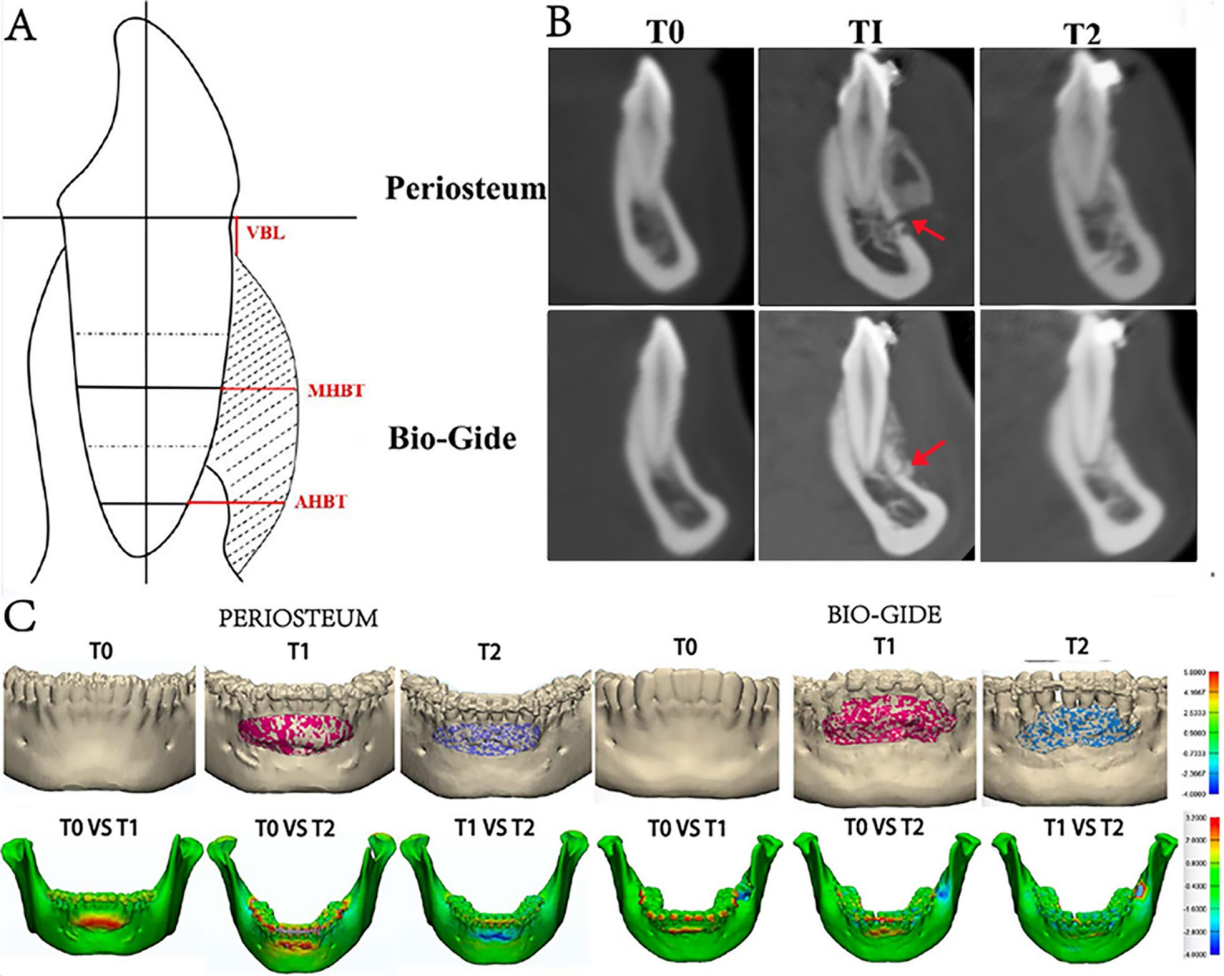
**A** Illustration of radiographic assessments; **B** CBCT images of the lower left canine for two groups at 3 different time points (red arrow indicates corticotomy); **C** 3D reconstruction and surface distances on color-coded maps of the lower anterior region measured preoperatively and 12 months after PAOO in the test and The comparator groups

The maximum labiolingual sections of the mandibular left canine were selected using Dolphin software (version 11.7, Chatsworth, Calif, USA). The 3D reconstruction and registration of longitudinal scans taken at different time points were performed using Mimics 18.0 software (Materialise, Leuven, Belgium). According to our previous study, VBL of the mandibular canine was defined as the distance between the coronal crest and the CEJ at the labial surface, parallelly to the long axis of the tooth. MHBT and AHBT was measured from the midpoint of the middle third (or apical third of the mandibular left canine to the limit of the labial cortical surface, respectively, perpendicularly to the long axis of the tooth (Fig. 3A) [14].

### Periodontal measurements

Periodontal parameters were assessed for the lower left canine in each patient at T0 and T2[22]: (1) probing depth (PD)was defined as the distance from the gingival margin to the bottom of the gingival sulcus using a William’s probe (mesial, distal, and midpoint for both the labial and lingual surfaces)[23]; (2) clinical attachment level (CAL) was determined by the distance from the CEJ to the base of the sulcus at 6 sites asmentioned [24] and (3) width of keratinized tissue (WKT) measured from the mucogingival junction to the free gingival margin evaluated mid-buccally [25].

### Statistical analysis

According to the intention to treat (ITT) principle, the method of ‘/baseline observation carried forward” was used to impute the missing data for participants’ measurements during follow-up where the data were missing. It ensures that all randomized patients are included in the analysis, thereby maintaining comparability between treatment groups at baseline and minimizing confounding [26]. The per protocol (PP) analysis was conducted for post-surgical complications.

All analyses were performed using statistical software package (SPSS, version 17.0, Chicago, Ill). Categorical data were presented as numbers and percentages and analyzed by chi-square test. Quantitative data were expressed as means with standard deviation. Non-parametric Mann–Whitney U-test was used for comparisons between groups. The Wilcoxon signed-rank test was used to evaluate the changes of post-operative complications and periodontal measurements in each group. Friedman's test was taken to compare radiological variables at different time points. All statistical hypothesis tests were two sided and a p value of less than 0.05 was regarded as statistically significant.

## Results

### Participates

**Fig. 4:**
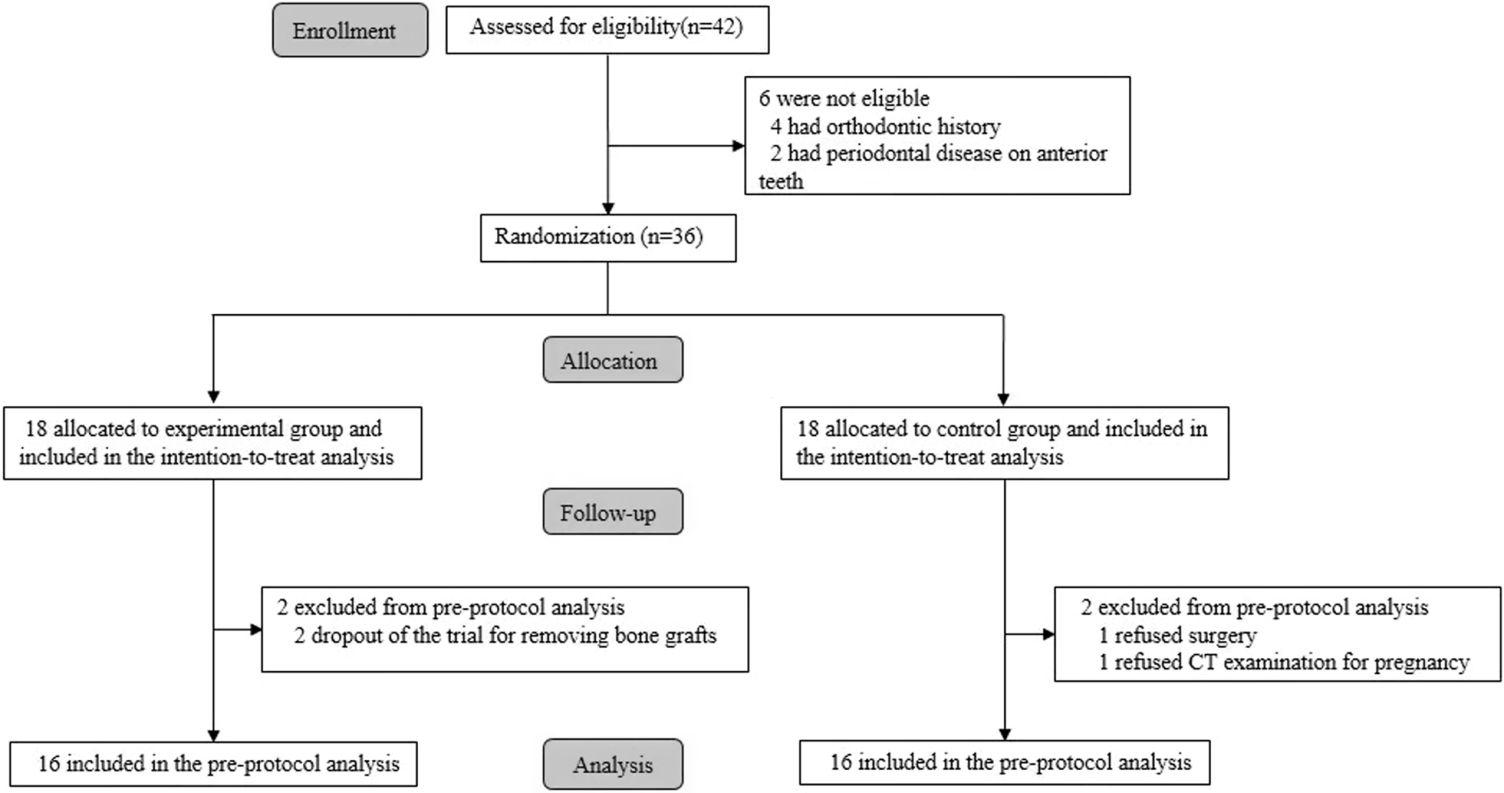
CONSORT flow diagram

From September 2017 to February 2018, 36 adult subjects were eligible and recruited in the present study. The follow-up period was 12 months after surgery. Except for soft tissue complications, no procedure- or device-related adverse events were observed in any patient. The CONSORT flowchart was displayed in Fig. 4. After random assignment, 1 patient in the comparator group refused the surgery. All the rest patients completed the full treatment protocol except that 1 patient with pregnancy and 2 patients with removal of bone grafts after surgery withdrew from the trial. This study was finally completed for 32 patients. No significant differences in characteristics were found between the two groups at baseline (Table 1).

**Table 1:** Baseline data of test and control group (intention to treat analysis)

	Test group	Control group	Between groups (*P*)
Age mean^†^	20.78 ± 2.24	21.06 ± 2.92	NS
*Sex, n (%)*^‡^			
Male	2(11.1)	4(22.2)	NS
Female	16(88.9)	14(77.8)	NS
*Malocclusion n, (%)*^‡^			
I	2(11.1)	2(11.1)	NS
II	9(50)	11(61.1)	NS
III	7(38.9)	5(27.8)	NS
Dehiscences, sites (%)^‡^	14(77.8)	12(66.7)	NS
Fenestrations, sites (%)^‡^	6(33.3)	4(22.2)	NS^‡^

†Mann–Whitney test

‡Chi-square test

### Operative time and postoperative complications

The mean operative time of the comparator group was significantly short than that of the tested group (*P* < 0.001) (Table 2). Facial swelling value, in both two groups, was much lesser at 7d postoperative as compared to 2d (*P* < 0.01). By contrast, no statistically significant difference was observed between 2 groups when postoperative swelling was evaluated at the early and later postoperative period. Pain values showed a significant reduction from the 2nd day to the 7th day after the surgery (*P* < 0.01) and there were no statistically significant differences between the 2 groups either at day 2 or at day 7 (Table 2).

**Table 2:** The operative time and post-operative complications of test and control groups (per protocol analysis)

	Test group, n = 16	Control group, n = 16	Between groups (*P*)
Time^†^	61.19 ± 4.53	33.87 ± 4.66	< 0.001
*Pain score (VAS)*^†^			
2d	4.63 ± 1.02	5.31 ± 1.2	0.160
7d	2.56 ± 0.89	3.34 ± 1.25	0.061
Within groups(*P*) ^‡^	< 0.001	< 0.001	
*Swelling (tragus-Pogonion, mm)*^†^			
2d	10.62 ± 4.8	9.94 ± 4.81	0.752
7d	4.87 ± 4.29	5.00 ± 6.27	0.381
Within groups(*P*)^‡^	0.004	0.005	
Infection (%)^§^	2(12.5)	0(0)	0.484
Numbness of the lip (%)^§^	1(6.25)	2(12.5)	1.000

†Mann–Whitney test

‡Wilcoxon signed-rank test

§Chi-square test

### Radiographic evaluations

Table 3 shows the differences in radiographic bone augmentation parameters between the 2 groups. At T2, the mean VBL value was 2.52 mm in the periosteum covered group, and 4.17 mm in the experimental group, with a significantly difference (*P* < 0.05). With respect to the VBL, a significant reduction was observed from T0 to T1 (*P* < 0.001) in both groups, and a significantly increase from T1 to T2 (*P* < 0.001) in the Bio-Gide group. However, the VBL at T2 was still significantly lower than that at T0 for both groups. Significant increases of MHBT and AHBT were observed at T1 and T2 compared with T0 in each group (*P* < 0.001). Taken all together, the results indicated bone augmentation could be accomplished in both groups. The experimental group, however, provided better results than the comparator group in consideration of the vertical level (Fig. 3B, C).

**Table 3:** Radiographic parameters of test and control groups over time (intention to treat analysis for the lower left canine)

	T0	T1	T2	Within groups (*P*)^†^
*VBL (mm)*				
Test group, n = 18	6.21 ± 1.54	2.13 ± 2.45	2.52 ± 2.23	< 0.001
Control group, n = 18	6.06 ± 2.91	1.36 ± 1.92	4.17 ± 2.6	< 0.001
Between groups(*p*)^‡^	0.963	0.279	0.027	
*MHBT (mm)*				
Test group, n = 18	0.13 ± 0.25	3.31 ± 1.58	1.95 ± 1.12	< 0.001
Control group, n = 18	0.17 ± 0.43	3.14 ± 1.79	2.18 ± 1.25	< 0.001
Between groups(*p*)^‡^	0.696	0.650	0.462	
*AHBT (mm)*				
Test group, n = 18	1.64 ± 0.57	4.29 ± 1.80	3.16 ± 2.18	< 0.001
Control group, n = 18	1.92 ± 0.97	4.05 ± 1.62	3.64 ± 2.22	< 0.001
Between groups (*p*)^‡^	0.864	0.650	0.501	

*T0* at baseline; *T1* postoperative 1 week; *T2* postoperative 12 months

†Friedman’s test

‡Mann–Whitney test

### Periodontal evaluations

No soft tissue defect occurred and the esthetics of soft tissue contour was improved during the follow-up. The periodontal parameters measured at two time points were reported in Table 4. No statistically significant differences were observed between the two groups with respect to PD, CAL and WKT at T0 or T2.

**Table 4:** Periodontal parameters of test and control groups over time (mm) (intention to treat analysis for the lower left canine)

	T0	T2	Within groups (*P*)^†^
*PD*			
Test group, n = 18	1.02 ± 0.50	1.02 ± 0.33	0.979
Control group, n = 18	1.05 ± 0.40	1.02 ± 0.55	0.815
Between groups(*p*)^‡^	0.815	0.938	
*CAL*			
Test group, n = 18	0.69 ± 0.85	0.53 ± 0.68	0.176
Control group, n = 18	0.81 ± 1.22	0.42 ± 0.42	0.777
Between groups(*p*)^‡^	0.308	> 0.999	
*WKT*			
Test group, n = 18	2.57 ± 0.47	2.94 ± 1.04	0.213
Control group, n = 18	2.86 ± 1.41	2.94 ± 1.36	0.274
Between groups (*p*)^‡^	0.888	0.913	

*T0* at baseline; *T2* postoperative 12 months

†Wilcoxon signed-rank test

‡Mann–Whitney test

### Harms

There was some damage done in the process of stripping periosteum during the surgery. Additionally, two patients suffered from wound infection on the recall 7 days and antibiotics administration combined with complete graft removal were carried out for these patients in the experimental group. Primary wound closure was achieved and maintained after the surgery in each group. Two patients in the experimental group and 1 patient in the collagen membrane covered group had numbness of the lip at 7d follow-up. These patients fully recovered within 4 weeks after neurotrophic drug treatment (Table 2).

## Discussion

The periosteum-covered PAOO demonstrated better outcome in vertical alveolar bone augmentation during the 12-month healing period. Based on a three-dimensional CBCT method, it was observed that more alveolus augmentation was achieved at the apical portion than the coronal portion and the vertical alveolar regeneration could not be achieved for the tradition PAOO treatment. The possible reason may be that soft tissue tension at the incision site would compromise the end result of the traditional PAOO therapy. The coronally repositioned flap after regenerative surgery might have compressed bone substitutes and decreased the original bone volume of alveolar crest. Another factor may be related to the displacement of bone grafting materials occurred during the healing period. Although collagen membrane is considered a biodegradable material with no need for removal, however, it is unable to fully maintain the augmented contour. This could be explained by an eventual membrane collapse or displacement after its application.

Radiographic results demonstrated periosteum-covered approach could be used for reconstruction of vertical alveolar defect and maintenance of the augmented vertical bone over time, which is in accordance with our previous study [14]. In the periosteum-fixed technique, the periosteum served as an encapsulating membrane to maintain bone substitutes in the desired position without displacement. As dehiscences were seen frequently in the mandibular anterior regions, vertical alveolar regeneration represents an essential outcome when treating alveolar defect underwent orthodontics [2, 27]. This novel PAOO technique permits to gain extensive bone augmentation, especially for vertical augmentation by complete periosteum coverage in a dumpling-like fashion. The high stability of the periosteum is associated with its tension-free coverage and served as a favourable fixation ensuring the immobilization of graft materials and effective bone regeneration. Although a skeletal anchorage device was reported to be applied in traditional PAOO procedure temporarily for space maintenance, unacceptable foreign body reactions and secondary surgical removal were potential problems with this technique [28].

Except for acting as a physical barrier, the periosteum has another advantage: an activator of vascular remodeling via close contact with the recipient site. It refers to that the periosteum could contribute to reconstruction of the vascular blood supply of the bone graft. Periosteum is a connective tissue membrane that includes a fibrous and a cambium layer. The latter layer consists the cellular components that facilitate bone remodeling and their precursor cells [29]. Abundant microvessels are distributed in the fibrous layer, which contains endothelial pericytes that can differentiate into many kinds of cell types, including osteoblasts. In addition, the periosteum plays a critical part in osteoclast resorption and early graft vascular reconstruction [29, 30]. Periosteal preservation significantly accelerated the formation of the new bone as compared with bone healing without periosteum [31].

In orthodontically treated patients, mandibular anterior teeth seem to be most vulnerable to the development of gingival recessions, especially in individuals with thin alveolar bone [32, 33]. We choose the mandibular left canine as our main objective for the following reasons: (1) the teeth most often affected by dehiscence were the mandibular canine [34]; (2) canine located at the corner of the dental arch increases the difficulty of alveolar augmentation. Successful bone grafting in this region could reflect the level of the surgical technique; (3) The clustering link to the same patient could be avoided. Nevertheless, the results of clinical parameters showed that these two treatment modalities did not compromise the periodontal health. The bone augmentation may act an important role in the maintaining the position and dimensions of the soft tissue.

Both techniques adopted in the present study exhibited several surgery complications. The periosteum-covered procedure needs delicate periosteum dissecting and holes drilling on the bone surface for suture fixation, which increases operating times and risks of postoperative complications occurring. It is worth noting that 2 patients had infection after this novel technique. The possible reason may be that when the periosteum was over-elevated at the alveolar crest, the bone graft area would be directly connected with the oral cavity, causing leakage of bone graft substitutes and inflammatory reaction. In addition, vertical alveolar regeneration may be limited by the attachment level of periosteum, thus this technique may be more indicated for alveolar defect without associated gingival recession. Long-term follow-up is also required to determine the stability of these results.

We can observe clear corticotomy line as well as bone substitutes placed on the original bone surface directly post grafting using CBCT images. It appears that grafting materials were mixed with good bony consolidation and uninterrupted bone cortex was formed on the outermost layers at 1 year postoperatively. The weakness of this study is that the biodegradation of grafting materials was not possible to evaluate in CT scans. Histological analyses would be recommended to solve this problem; however, bone biopsy has been a major obstacle in this study. Other than that, the acquisition of periosteum is not always as good as it should be. However, certain surgical operations can improve the success rate of peeling the periosteum.

## Conclusion

The current study indicated that both periosteum-covered and bioresorbable membrane-covered PAOO regenerative procedures are effective in creating favorable alveolar conditions for orthodontic treatment. Nonetheless, in case of dehiscence with a higher prevalence in the mandibular anterior region, PAOO with periosteum coverage may be more indicated because more vertical bone augmentation could be achieved by this technique.

## Supplementary Information

**Additional file 1**. Basic patient information and dehiscence and fenestration data.

## Data Availability

All data generated or analysed during this study are included in this published article (and Additional file 1).
